# Stakeholder perspectives on the use of pig meat inspection as a health and welfare diagnostic tool in the Republic of Ireland and Northern Ireland; a SWOT analysis

**DOI:** 10.1186/s13620-016-0076-3

**Published:** 2016-11-07

**Authors:** C. Devitt, L. Boyle, D. L. Teixeira, N. E. O’Connell, M. Hawe, A. Hanlon

**Affiliations:** 1 School of Architecture, Planning and Environmental Policy, University College Dublin, Dublin, Ireland; 2 Teagasc Pig Development Department, Animal and Grassland Research and Innovation Centre, Moorepark, Fermoy, Co., Cork, Ireland; 3 Laboratório de Etologia Aplicada, Universidade Federal de Santa Catarina, Florianópolis, Brazil; 4 Institute for Global Food Security, Queens University Belfast, Northern Ireland Technology Centre, 18-30 Malone Road, Belfast, BT9 5BN UK; 5 College of Agriculture, Food and Rural Enterprise, Greenmount Campus, Tirgracy Road, Antrim, BT41 4PS UK; 6 School of Veterinary Medicine, University College Dublin, Belfield, Dublin 4 Ireland

**Keywords:** Pig health and welfare, PIGWELFIND, Producer communication, SWOT analysis, Stakeholder interviews

## Abstract

**Background:**

A SWOT (Strengths, Weaknesses, Opportunities and Threats) analysis is a strategic management tool applied to policy planning and decision-making. This short report presents the results of a SWOT analysis, carried out with *n* = 16 stakeholders i) involved in the pig industry in the Republic of Ireland and Northern Ireland, and ii) in general animal welfare and food safety policy areas. As part of a larger study called PIGWELFIND, the analysis sought to explore the potential development of pig meat inspection as an animal welfare and diagnostic tool.

**Findings:**

The final SWOT framework comprised two strengths, three opportunities, six weaknesses, and five threats. Issues around relationships and communication between producers and their veterinary practitioner, processors and producers were common to both the strengths and weakness clusters. Practical challenges within the processing plant were also named. Overall, the SWOT framework complements results reported in Devitt et al. (Ir Vet J 69:2, 2016) regarding problematic issues within the current system of information feedback on meat inspection especially within the Republic of Ireland, and the wider challenges of communication and problems of distrust.

**Conclusion:**

The results of the SWOT analysis support the conclusions from Devitt et al. (Ir Vet J 69:2, 2016), that trust between all stakeholders across the supply chain will be essential for the development of an effective environment in which to realise the full diagnostic potential of MI data. Further stakeholder engagement could seek to apply the findings of the SWOT analysis to a policy Delphi methodology, as used elsewhere.

## Findings

There is growing acknowledgement of the value of expanding the role of meat inspection (MI) to better inform animal health and welfare management plans on pig farms [1–4]. However, the realisation of the full potential of MI data as a health and welfare diagnostic tool can hinge on wider contextual issues such as stakeholder engagement and positive and open communication processes between processors and producers [5]. Building on earlier social science research detailed in Devitt et al. [5], this short report presents the stakeholder perspectives on the potential development of MI as an animal health and welfare diagnostic tool as part of a larger study called PIGWELFIND. The perspectives draw from stakeholders involved in the pig industry across the Republic of Ireland (ROI) and Northern Ireland (NI), and representatives from general animal welfare and food safety policy at a national and international level. Semi-structured telephone interviews with 13 pig producers, the results of which are reported elsewhere [5], were also conducted.

## Material and methods

The Consolidated Criteria for Reporting Qualitative Research (Coreq-32) checklist was used to ensure quality control in the study design, and analysis and reporting of data [6]. Participants were selected using a purposive sampling technique [7], and recruited with the assistance of third party individuals. In total, 16 participants were involved in this component of the study. Data collection took place in early 2014. Two focus groups were conducted: Focus Group 1 (FG1) with four ROI government veterinarians involved in meat inspection and farm animal welfare, and Focus Group 2 (FG2) with four NI meat inspectors. A further three telephone interviews were conducted with managers of pig processing plants (P1, P2, and P3). Individual face-to-face and telephone interviews were conducted with representatives from Bord Bia (the Irish state agency for the promotion of Irish food) (Pol1)), the Food and Veterinary Office (Pol2), Irish Department of Agriculture, Food and the Marine (Pol3), European Food Safety Authority (Pol4), and the Department of Agriculture and Rural Development NI (Pol5). All focus groups and interviews were audio-recorded, transcribed and anonymised.

The focus and objective of data collection was on identifying the internal Strengths and Weaknesses directly and indirectly related to the process of meat processing and inspection, and the external Opportunities and Threats (SWOT) to the development and use of MI data as an animal health and welfare diagnostic tool. A SWOT analysis is a strategic management matrix tool that can be useful to policy makers for the purpose of policy development, implementation and resource use [8]. Use of the SWOT framework can be found elsewhere. For example: in the use of MI in surveillance of poultry health and welfare [9]; in an overview of animal welfare standards and initiatives in the European Union [10]; in the application of methods to assess dairy cow welfare [11] and veterinary dairy herd health management [12]; and finally, in determining opportunities and constraints in the Irish dairy sector [13]. The key issues identified were structured and clustered according to whether or not they made a positive or negative contribution to the development of MI, and whether or not they were external or internal to pig slaughter processes [14].

## Results

Table 1 displays the SWOT framework, and details the key issues identified under each cluster.

**Table 1:** Framework showing the Strengths, Weaknesses, Opportunities and Threats regarding the use of pig meat inspection as a health and welfare diagnostic tool in the Republic of Ireland and Northern Ireland

Strengths	Weaknesses
• Regular and open communication between some processors and producers (P1, P3, Pol2, Pol4) • Potential scope at ante-mortem for welfare inspection and data gathering (P1, P2, FG2)	• Inconsistent and lack of feedback between processors and producers (P1, P2, P3, Pol3) • Poor relationships between some producers and processors (FG1 and 2, P1-3, Pol2, Pol3, Pol5) • Poor communication between veterinarians in the processing plants and private veterinary practitioners at farm level (FG1) • Problems of consistency in recording approaches at meat inspection, including what is being recorded, and terminology used to record certain conditions (P1, P3, FG1, FG2) • Processing related barriers including line speed, ability to record accurately and objectively, and ability to record multiple indicators and the severity of health and welfare issues (FG1, FG2, Pol3, P1, P2) • Poor follow-up to cross-border communication on health and welfare concerns (FG2)
Opportunities	Threats
• Positive relationships and interaction between producers and their private veterinary practitioner (FG1, 2, P1, P2, P3, Pol1, Pol3) • Learning from existing herd health related interventions (such as Northern Ireland’s Pig Grading Information System, and the Carcass Inspection Analysis software) (Pol5, P3, FG2) • Producer identity as business oriented and innovative (Pol1, 2, 4, 5)	• Gaps in cross-border communication on health and welfare concerns (FG2) • Lack of buy-in and cooperation across all stakeholders (Pol1-5) • Producers not being open to receiving external advice and support (FG1-2, P1-2, Pol3) • Producers’ view that diagnostic tool is form of surveillance (FG1, FG2, P1-3, Pol3, Pol5) • Producer distrust and a “them versus us” approach (FG1-2, P1-2, Pol3) • Producer attitudes towards pig health and welfare issues, which may undermine welfare standards (P1, P2, FG1, Pol2, Pol5) • Limited funding for development of diagnostic tool (Pol3)

A greater number of weaknesses (six) and threats (five) were identified than strengths (two) and opportunities (three), reflecting some of the challenges identified in Devitt et al. [5] pertaining to the development of MI.

## Discussion

A review of strengths and weaknesses suggest that issues of relationships and communication are central to the development of MI as a health and welfare diagnostic tool. Stakeholders recalled a number of instances of regular and open communication between producer and processors, and the benefit of this for producers’ sense of trust in processors, and in terms of their awareness of pig health and welfare issues. Stakeholders also identified as an opportunity, the value of existing relationships between producers and their private veterinary practitioner, especially in terms of working with producers on health and welfare concerns and visiting the processing plant on behalf of the producer. This point supports evidence from elsewhere on how veterinarians form a key component in facilitating effective communication, and capacity building at the farm level [5, 15–18]. Indeed, veterinarians could play an important role in knowledge transfer on issues such as tail biting and tail docking [19], in enabling the “dialogue process” identified by Benard et al. [20] as being important in helping farmers address complex welfare issues, and in directing producers to avail of advisory services, to help lower the risk of tail biting [21].

Identified weaknesses such as inconsistent, and a lack of, information feedback on meat inspection between processors and producers, and poor relationships and communication between industry actors, echo the key issues also identified by Devitt et al. [5]. In their study, interviewed producers, particularly those in the Republic of Ireland, expressed dissatisfaction regarding the perceived lack of detail and perceived inconsistency of information feedback received from pig-meat processors. This dissatisfaction amplified issues of distrust among producers, of processors [5, 22, 23] – an issue previously cited as a major problem for the development of the pig industry in the Republic of Ireland (ROI) [23].

In terms of the practical implications of the development of MI as a diagnostic tool, recording data on pig health and welfare, ante-mortem was considered by stakeholders as more achievable than post-mortem (i.e., on the carcass). Stakeholders attributed this ease to the relative absence of time constraints and better visibility of the pigs, although in reality this is not often the case [24]. However, while there is value in data collected at ante-mortem particularly relating to animals requiring special attention (or casualty animals), the key information on the health and welfare of the herd must be gleaned from the carcass and viscera post mortem (i.e., at MI) [4]. Unfortunately, this is where many barriers, or weaknesses, were identified including differences in recording approaches, line speed and resulting time limitations affecting how MI information is recorded. Additionally, difficulties were reported with recording issues such as severity of tail-biting because of the lack of well-defined scoring systems, and the absence of a means of ensuring that consistent terminology is used between meat inspectors, to describe the condition of the carcass.

Threats identified in the current study, included gaps and a lack of follow-up in cross-border communication on health and welfare issues, and concerns regarding producer, processor and policy buy-in and support for the use of MI as a diagnostic tool. These results support the contention that proper communication is central to stakeholder relationships [25–27], and that positive relationships and buy-in between industry actors are central to the development and utilisation of MI as a health and welfare diagnostic tool. Although producers were regarded as generally innovative and business oriented, concerns were expressed regarding producers not always being open to receiving advice and support from external sources, such as industry and government – reflecting similar findings by Hernández-Jover et al. [28, 29]. Furthermore, concerns over producer attitudes, towards pig welfare issues, mirrors results presented in Devitt et al. [5] regarding tolerance of potential welfare risk issues on-farm. To reiterate earlier comments, private veterinary practitioners can help better inform these attitudes.

Overall, the SWOT framework complements key results reported in Devitt et al. [5] regarding problematic issues within the current system of information feedback on meat inspection especially within the Republic of Ireland, and the wider challenges of relationships and communication, distrust and fairness concerns. As noted by Devitt et al. [5], these issues are not specific to the pig meat industry. However, overall, the results of the SWOT analysis support the conclusions from Devitt et al. [5], that trust between all stakeholders across the supply chain will be essential for the development of an effective environment in which to realise the full diagnostic potential of MI data.

## Conclusion

There are a number of limitations to the study. Stakeholders were not provided with the opportunity to review or comment on the SWOT framework. A workshop bringing all stakeholders together may have provided an opportunity for further discussion and review on each of the points presented in Table 1. Nevertheless, the range of perspectives and positions represented among the stakeholders contributed to a representative and diverse SWOT framework. A key limitation of the SWOT analysis used in this study is that the importance or impact of each factor was not measured quantitatively. This presents difficulties when aiming to determine which issues should be prioritised in future strategic planning. Further stakeholder engagement could seek to apply the findings of the SWOT analysis to a policy Delphi methodology, such as that applied by More et al. [30], although with an emphasis on identifying priorities for advancing the development of MI as a diagnostic tool. Nevertheless, a key finding of this research is that there is an urgent need to address the poor and inconsistent communication between stakeholders, which fosters a climate of distrust and may potentially impede the development of MI data as a pig health and welfare diagnostic tool.
